# Forest fire prediction using image processing

**DOI:** 10.1371/journal.pone.0338794

**Published:** 2026-01-20

**Authors:** Yingdan Li, Junting Chen, Yaxuan Zeng, Yuanyuan Ding, Chaobing Huang, Hongxing Tian

**Affiliations:** 1 School of Electronic Information Engineering, Guiyang University, Guiyang, China; 2 School of Computer Science and Engineering, University of Electronic Science and Technology of China, Chengdu, China; 3 Fire Brigade, Ziyun County, Anshun, China; Xi'an Jiaotong-Liverpool University, CHINA

## Abstract

Forest fires pose a significant threat to public safety and the environment, and harmful pollutants spread rapidly in areas covered by vegetation. Early detection is very important for preventing forest fires from evolving into catastrophic fires. The traditional prediction methods have relatively low accuracy. They can only identify fires clearly after they occur, making it difficult to meet the requirements of precise real-time detection. The YOLOv5-PSG model proposed in this paper improves the YOLOv5 model. After 300 rounds of training, the average recognition accuracy rate of mAP can reach 93.1%, and the accuracy rate can reach approximately 0.802. After 300 rounds of training and learning, the confidence level can reach about 0.965. This improvement makes fire early warning and prediction more comprehensive and effective, ultimately protecting human life and the environment by mitigating the impact of wildfires.

## 1 Introduction

Fires have a significant impact on the daily lives of people. In 2023, global wildfires burned 384 million hectares (Mha), which is the highest recorded since 2017. These fires emitted an estimated 2524 × 10^12^ grams of carbon (Tg C) [1]. It is evident that fire not only endangers humans and animals and results in considerable economic losses to society but also affects the development of the ecological environment and social stability. This issue is of great significance. To protect people’s safety and property, it is important to identify fire sources in the early stages of a fire [2] and to prevent the spread of those sources.

In the past, familiar fire source detection methods were generally implemented using detectors that sense smoke, temperature, and light sources [3]. This method can only detect the degree of fire development [4] and cannot identify or provide feedback during the early stages of fire source occurrence. Moreover, it has a certain false alarm rate and is easily influenced by environmental factors, such as dust, smoke, water fog, high-temperature weather, and other forms of interference. This interference can lead to false alarms, necessitating regular checks by a certain amount of manpower [5].

With the continuous maturation and promotion of artificial intelligence technology, mature artificial intelligence algorithms have been applied in various industries.Kemal Akyol et al. developed an innovative artificial intelligence model for detecting forest fires from landscape images [6].This study proposes a hybrid approach that fuses Transformer-based deep features extracted from architectures such as BEiT, ViTHybrid [7], and SwinV2 with deep neural networks (DNNS) for classification [8]. The research aims to meet the demand for accurate and rapid fire detection in order to minimize the damage caused by forest fires.

Mounia Aaricha et al. focused on conducting research on forest fire detection and prediction by using deep learning methods and with the aid of satellite images [9]. The paper first introduces a variety of deep learning methods commonly used for forest fire detection, such as convolutional Neural Network (CNN), U-Net, InceptionV3 [10–12], etc. Then, the commonly used satellite image datasets in this field were sorted out, such as Sentinel-2, Landsat-8, MODIS, etc. These datasets have played an important role in forest fire detection. Finally, based on the existing research results, a comparative study was conducted on the performance of different deep learning methods in forest fire and smoke detection and prediction. By comprehensively analyzing the advantages and disadvantages of various methods and datasets, it provides a reference for subsequent research in selecting appropriate satellite images, constructing robust datasets, and improving deep learning methods.

Azlan Saleh et al. studied deep learning-based forest fire monitoring systems [13]. They analyzed 37 papers from 2018–2023, covering data types, augmentation methods, and model architectures. The research divided deep learning applications into five subfields and evaluated models using multiple indicators. Most models showed over 90% accuracy. The paper pointed out issues like scarce training data and dataset imbalance, and suggested optimizing hyperparameters, integrating multimodal satellite data, using generative augmentation, and refining model architectures to improve detection systems.

Ting Yun, Jian Li et al. systematically reviewed the current situation, application and prospects of deep learning technology in forest research in their paper, covering deep learning methods for various types of forest remote sensing data (such as images, point clouds, and fused data). The deep learning methods were classified based on data processing methods and operating principles. And the diverse applications of it in the forest were classified as examples [14]. The study also summarized the main forest-related datasets and analyzed the global geographical distribution of relevant literature in the past five years. Meanwhile, the advantages and limitations of the existing deep learning technologies in forest research were comprehensively sorted out, and the development directions of applying deep learning technologies in fields such as forest phenotype analysis, carbon storage estimation, and digital twin creation in the future were prospected, aiming to provide comprehensive references for scholars and industry experts in related fields.

In the algorithm based on “You Only Look Once” (YOLO), scholars such as Shikuan Wang proposed the innovative algorithm FireSmoke-YOLO for the detection problems of complex fire scenarios and small scattered targets [15]. This study is based on the YOLO framework. By introducing the Funnel Spatial pyramid pooling fast layer (FSPPF), the small object detection layer and the dynamic serpentine convolution (DSC), the feature extraction and fusion capabilities are optimized, and a comprehensive dataset containing 11,300 multi-scene images is constructed to improve the generalization of the model. Experiments show that the mAP50 of this model reaches 81.4% and the MAP50-95 reaches 59% [16]. It has both high precision and lightweight (56.1 MB), shows strong generalization ability for satellite remote sensing data, provides an efficient solution for fire monitoring, and has wide application potential in the fields of fire protection and environmental protection.

The structure of this article is as follows: Section 2 introduces our YOLOv5-PSG algorithm, explains the improvement of its network structure model, as well as the design and training strategies of the loss function. The subsequent sections 2.2 to 2.4 cover the construction, annotation and preprocessing of the dataset, as well as the details of the metrics. Section 3 introduces the influence of training rounds on the results. The subsequent sections 3.2 to 4.4 respectively analyze the experimental results, conduct ablation experiments on our model, and compare it with the classic algorithms of the YOLO series, analyzing the key differences of each algorithm. Finally, Section 5 presents the conclusion, summarizes the key points of this paper, and conducts a summative assessment of the potential and limitations of YOLO in fire detection.

## 2 Materials and methods

**Fig 1:**
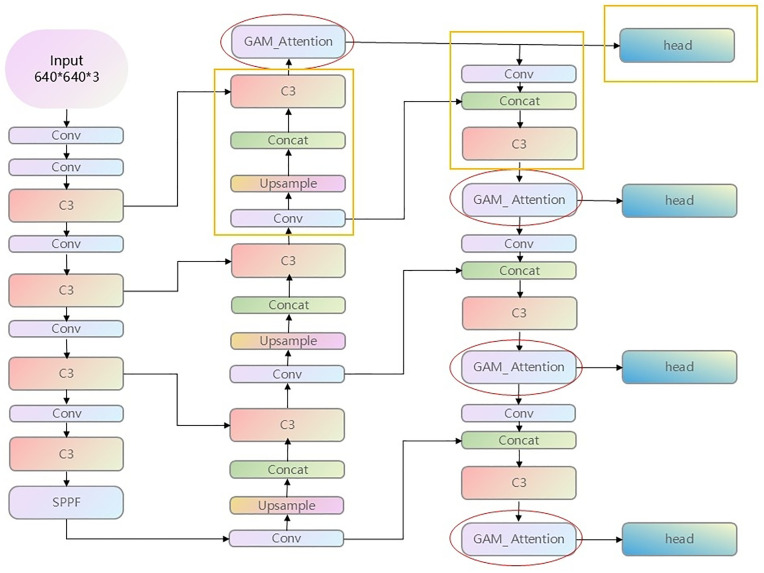
YOLOv5-PSG network structure diagram.

This paper first optimizes the branch sampling of small targets. Small targets usually occupy a very small pixel area in an image and are easily overwhelmed by the large-scale features on the high-level feature map. By adding an additional detection head to the shallow feature map, more local information can be utilized, thereby improving the detection accuracy of small targets. This detection head will perform detection on a finer feature map (i.e., a feature map with higher resolution) in order to better capture small targets. The framework of the YOLOv5-PSG model proposed in this paper is shown in Fig 1. In the yellow box part, in the Neck part of the basic YOLOv5, a new Conv-Upsample-Concat-C3 is added after the original head output of the 18th layer for small target branch sampling. Then, a Conv-Concat-C3 structure is used for the convolution of small target branches, and a detection head output is added.

In the task of fire target detection, the loss function is used to measure the difference between the model prediction results and the real labels. The loss function is generally composed of classification loss, regression loss and confidence loss. Suppose the model has N prediction boxes and C categories. The loss function L of the improved YOLO model can be expressed as Formula (1):


$$\;\;\;L_{PSG}=\lambda_{cls}L_{cls}+\lambda_{reg}L_{reg}+\lambda_{obj}L_{obj}$$ (1)

Among them $\lambda_{cls}$, $\lambda_{reg}$ and $\lambda_{obj}$ are weight coefficients, which are respectively used to balance the contributions of classification loss, regression loss, and confidence loss.

Classification Loss usually adopts Cross-Entropy loss. For each prediction box, calculate the cross-entropy between the probability distribution of its predicted category and the true category label. The classification loss function is as shown in Formula (2).


$$\begin{array}{c} L_{cls}=-\frac{\text{1}}{N}\sum_{i=\text{1}}^{N}\sum_{c=\text{1}}^{C}y_{i,c}\;\text{log}\left({\hat{y}}_{i,c}\right)+\left(\text{1}-y_{i,c}\right)\text{log}\left(\text{1}-{\hat{y}}_{i,c}\right) \end{array}$$ (2)

Among them, $y_{i,c}$ is the true label (0 or 1) of category c in the i-th prediction box, and ${\hat{y}}_{i,c}$ is the probability of predicting the category c in the i-th prediction box.

In the task of fire target detection, the loss function is used to measure the difference between the model prediction results and the real labels. The IoU loss function is used to measure the degree of overlap between the predicted box and the true box. This model adopts the CIoU loss function, as shown in Formula (3):


$$L_{reg}=\frac{\text{1}}{N}\sum_{i=\text{1}}^{N}\left(\text{1}-CIoU(b_{i},{\hat{b}}_{i})\right)$$ (3)

Among them, $b_{i}$ is the coordinate of the i-th true bounding box (usually the center coordinate (x,y) and the width and height (w, h)), and ${\hat{b}}_{i}$ is the coordinate of the i-th predicted bounding box, which is the complete intersection-union ratio.The calculation formula of CIoU is Formula (4).


$$CIoU=IoU-\frac{\rho^{\text{2}}(b,b^{gt})}{c^{\text{2}}}-\alpha v$$ (4)

Among them, IoU is the intersection and union ratio, $\rho^{\text{2}}(b,b^{gt})$ is the square of the distance between the center points of the prediction box and the real box, c is the diagonal length of the smallest circumscribed rectangle that contains the prediction box and the true box, α is the weight coefficient, and v is the parameter for measuring the consistency of the aspect ratio.

In the confidence loss $L_{obj}$, the confidence level represents the probability of whether there is a target in the prediction box. The binary classification cross-entropy loss is also adopted, such as formula (5):


$$\begin{array}{c} L_{obj}=-\frac{\text{1}}{N}\sum_{i=\text{1}}^{N}y_{i,obj}\;\text{log}\left({\hat{y}}_{i,obj}\right)+\left(\text{1}-y_{i,obj}\right)\text{log}\left(\text{1}-{\hat{y}}_{i,obj}\right) \end{array}$$ (5)

Among them, $y_{i,obj}$ is the true label (0 or 1) of the existence of the target in the i-th prediction box, and ${\hat{y}}_{i,obj}$ is the probability of the existence of the predicted target in the i-th prediction box.

When we use deep models to recognize images, we usually extract the local information of the image through convolution kernels. However, the influence of each local information on whether the image can be correctly recognized is different. How to make the model know the importance of different local information in the image, we introduce the attention mechanism here. The YOLOv5-PSG model proposed in this paper has a red elliptical part. Before each detection head, an attention layer GAM_Attention module is added.

The output of GAM_Attention is the product of the original feature and the attention weight, as shown in Formula (6):


$$F^{\prime }=F⊗M_{c}(F)⊗M_{s}(F)$$ (6)

Here, ⊗ represents element-by-element multiplication (Hadamard product, that is, the product of elements at the corresponding position; F represents the original feature map input into the GAM_Attention module, which is the feature information extracted by the model before this module; $M_{c}(F)$ is the channel attention weight matrix output by the channel attention module. It is obtained through operations such as global average pooling, linear transformation, and activation on the input feature map F, reflecting the importance of different channels; $M_{s}(F)$ is the spatial attention weight matrix output by the spatial attention module. It is obtained through operations such as convolution and activation on the input feature map F, reflecting the importance of different spatial positions of the feature map; $F^{\prime }$ is the output feature map processed by the GAM_Attention module, which is the result of weighting the original feature map F with the channel attention weight $M_{c}(F)$ and the spatial attention weight $M_{s}(F)$.

During the training process, the total loss function of the model is the sum of the cross-entropy loss and the regularization term, as shown in Formula (7);


$$L=L_{\text{PSG}}(f_{\theta }(F^{\prime }),y)+\lambda \cdot \Omega (\theta )$$ (7)

Among them: $L_{\text{PSG}}$ is the task loss function, which is the binary cross-entropy loss; $f_{\theta }$ is the model with GAM_Attention; y is the true label; Ω(θ) is the regularization term; λ is the hyperparameter of the regularization strength.

### 2.1 Experimental plan

**Fig 2:**
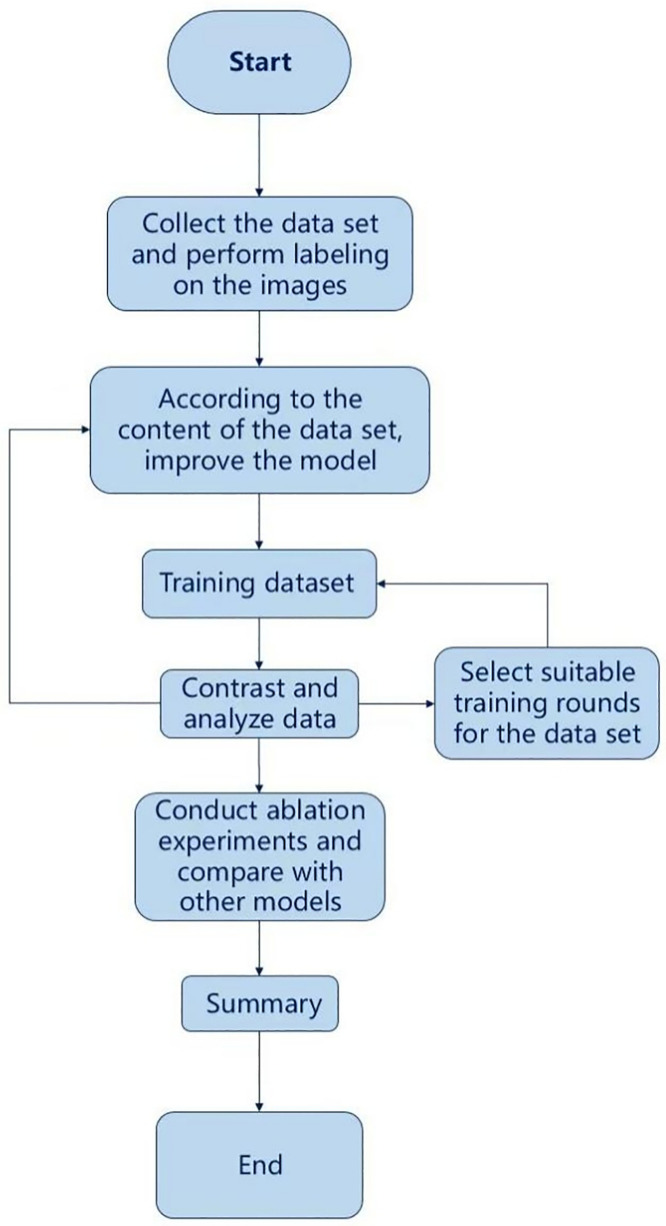
System design drawing.

Compared with YOLOv3, YOLOv5 [17] features a more optimised network structure, fewer parameters, a more lightweight model, faster detection speed and a more flexible recognition mode, allowing it to identify many images in a short time. In addition, YOLOv5 uses the PyTorch framework [18] combined with artificial intelligence vision technology [19], making it very convenient for users to train their own datasets. Therefore, YOLOv5 has been chosen as the basic framework for detecting and identifying high-altitude forest fire sources in this project. The specific system design is shown in Fig 2.

### 2.2 Description of the experimental dataset

#### 2.2.1 Overall overview of the experiment.

In the early stage of the experiment, 2,400 images of smoke from high-altitude forest fire sources and 600 images of candlelight were collected for comparison. The preliminary experimental results show that the recognized mAp50 is 0.675. Due to its poor prediction effect of fire source smoke, most of the smoke cannot be accurately identified. The environment in most fire source images is relatively complex and blurred, resulting in poor recognition effect of fire sources. Moreover, the performance of the model trained on small datasets is not satisfactory. To improve the experimental effect, we expanded the dataset and added 18,585 images of high-altitude forest smoke, bringing the total number of images to 21,585 [20]. We used the segmentation script to randomly extract one-tenth of the images from the 21,585 image dataset and began to conduct comparisons of 50, 100, 200, 300, 400 and 500 rounds of training. This work selects 300 as the optimal number of training rounds, optimizes the training model, and compares and analyzes the training graph results under the running directory. We studied and optimized the number of training rounds to determine whether the quality of the weighted document dataset could be improved, thereby enhancing the confidence level of image recognition [21].

#### 2.2.2 Collection of datasets.

**Fig 3:**
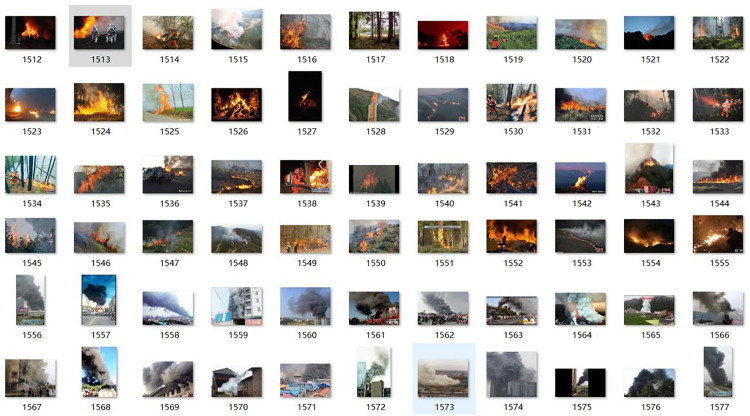
Fire source smoke dataset.

Finally, 21585 unified images of high-altitude forest fire source smoke and horizontal fire source smoke were selected in ascending order from Baidu Images and the Kaggle data platform to serve as the dataset for this experiment.As shown in Fig 3.

**Fig 4:**
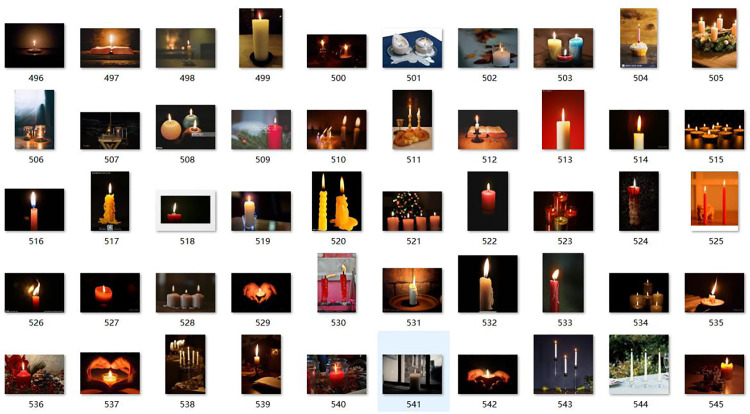
Candlelight dataset.

Moreover, 5. Additionally, 600 candle images were collected to analyze the firelight, as shown in Fig 4.To improve the effectiveness of model training, the first 600 images in the dataset consist of candlelight images, whereas the last 20985 images in the dataset comprise high-altitude fire source smoke images and horizontal fire source smoke images.

#### 2.2.3 Dataset annotation.

**Fig 5:**
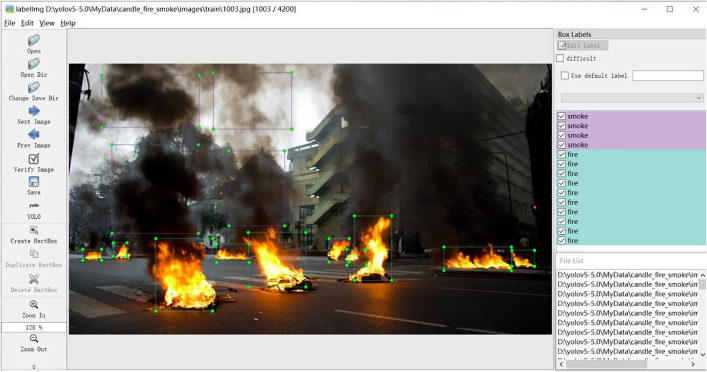
Target annotation page.

Open-source software was used to select and name the rectangular frames of the pictures, labelling each one individually.As shown in Fig 5.

### 2.3 Training environment

The operating system of the hardware platform is Windows 11 Professional Edition, Intel(R) Core(TM) i9-10900K CPU @ 3.70GHz, 64G, GeForce RTX 3090 24G, and the encoding platform is Pycharm, Python3.11. The main frameworks of deep learning are Pytorch and CUDA 12.4.

The total number of iterations for network training is 300 rounds. The initial learning rate is set at 0.01, and the final learning rate is set at 0.01. The first three rounds of training adopt Warmup preheating training, and the subsequent training uses the cosine annealing algorithm to stabilize the attenuation rate.

### 2.4 Evaluation index

In this paper, the performance of the algorithm is evaluated by using evaluation metrics such as Precision (P), recall rate (R), F1 score, AP(Average Precision), mAP (mean of Average Precision), and Frames Per Second (FPS). Accuracy is used to measure the correctness of the algorithm in all samples predicted as fires. The recall rate represents the ability of an algorithm to correctly identify fire samples. The F1 score is the harmonic mean of precision and recall rate. The frame rate per second represents the number of images detected per second and is used to evaluate the detection speed of the algorithm. AP is the average accuracy of a single category, reflecting the robustness and generalization ability of the model. mAP calculates the average value of aps for all categories. AP is the average accuracy of a single category, reflecting the robustness and generalization ability of the model. mAP calculates the average value of aps for all categories. The calculation formulas are shown in Equations (8)–(13).


$$P=\frac{\text{TP}}{\text{TP}+\text{FP}}$$ (8)

$$R=\frac{TP}{TP+FN}$$ (9)

$$F\text{1}=\frac{\text{2}\times P\times R}{P+R}$$ (10)

$$FPS=\frac{\text{1}}{Processing\;time\;per\;fr\text{a}me}$$ (11)

$$\text{A}P=\int_{\text{0}}^{\text{1}}P(r)dr$$ (12)

$$m\text{A}P=\frac{\sum_{i=\text{1}}^{K}{AP}_{i}}{K}$$ (13)

TP represents the number of samples that are actually positive and correctly predicted to be positive. FN represents the number of samples that are actually in the positive class but are predicted to be in the negative class; FP represents the number of samples where the negative class is wrongly predicted as the positive class; TN represents the number of samples that are actually negative classes and correctly predicted to be negative classes, and K represents the total number of classes checked by the model.

## 3 Experiment and result analysis

### 3.1 Image visualisation renderings

**Fig 6:**
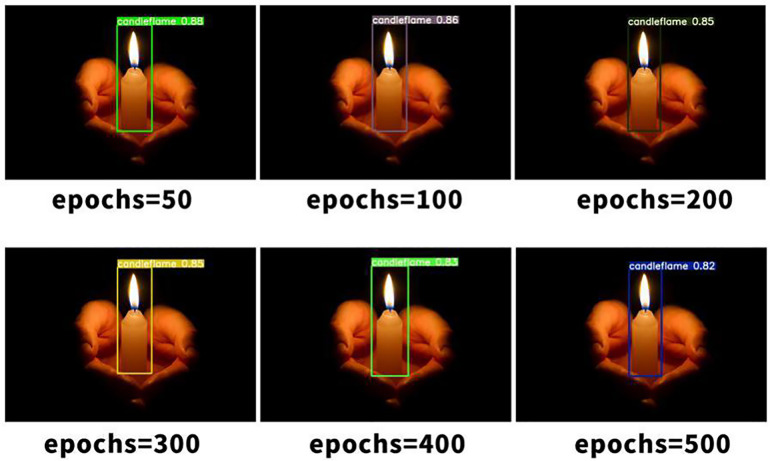
Visual comparison of candlelight.

**Fig 7:**
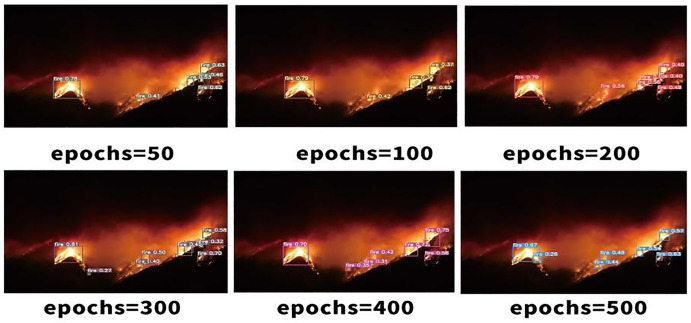
Visual comparison of high-altitude forest fire sources in complex scenes.

**Fig 8:**
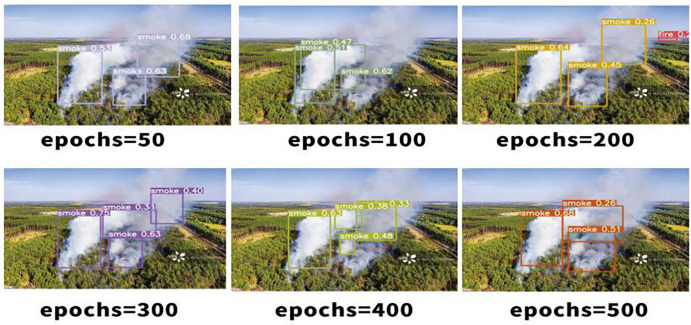
Comparison of high-altitude smoke visualisations.

The separated verification set and dataset were trained, with the number of training rounds set at 50. Additionally, 100, 200, 300, 400 and 500 rounds were stacked successively. A comparison of the visualisation effects of smoke and candlelight from high-altitude forest fire sources is shown below.As shown in Figs 6–8.

**Fig 9:**
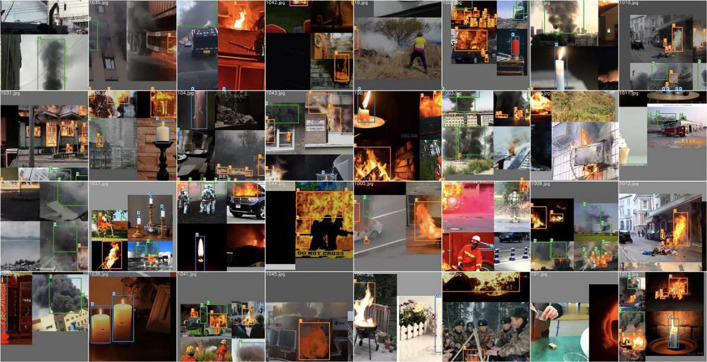
Diagram of data enhancement using arbitrary scaling cuts [20].

**Fig 10:**
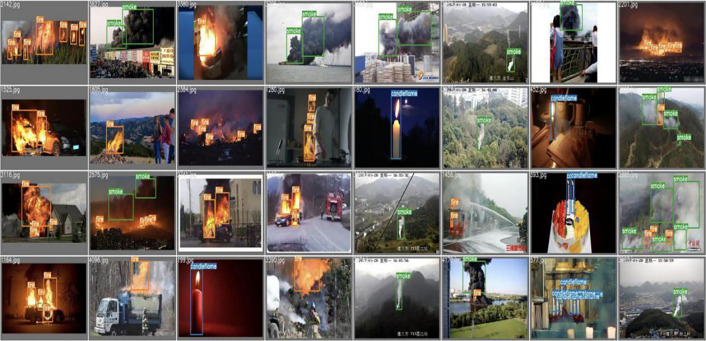
Diagram of positioning picture categories [20].

**Fig 11:**
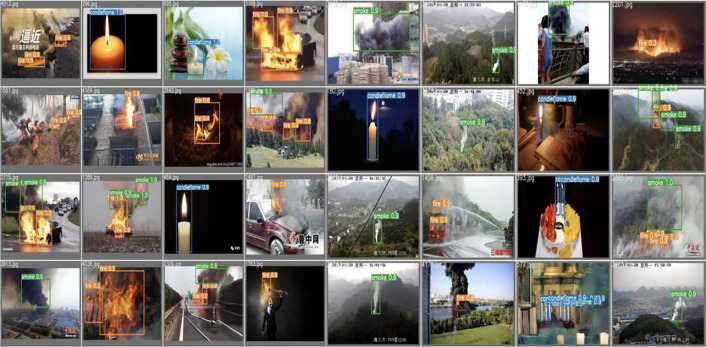
Confidence graph for test pictures [20].

The comparison of the effects of different training rounds shown above reveals that when the number of training rounds reaches 300, the confidence degree of smoke detection [22] for high-altitude forest fire sources is at its highest. When the number of training rounds is either less than or greater than 300, the confidence degree of fire source smoke recognition decreases. However, the confidence degree is greater when the number of training rounds for target recognition [23] is 50 or less, compared with when the number of training rounds involves simple scenes, such as candlelight. The following is an analysis of the training results for high-altitude forest fire source detection under the run catalogue. The category recognition of the images in the test set is generally accurate, as shown in the comparison between Figs 9–11, with the confidence degree being at or above 0.9 for clear images of the fire source.

### 3.2 Analysis chart of training results

**Fig 12:**
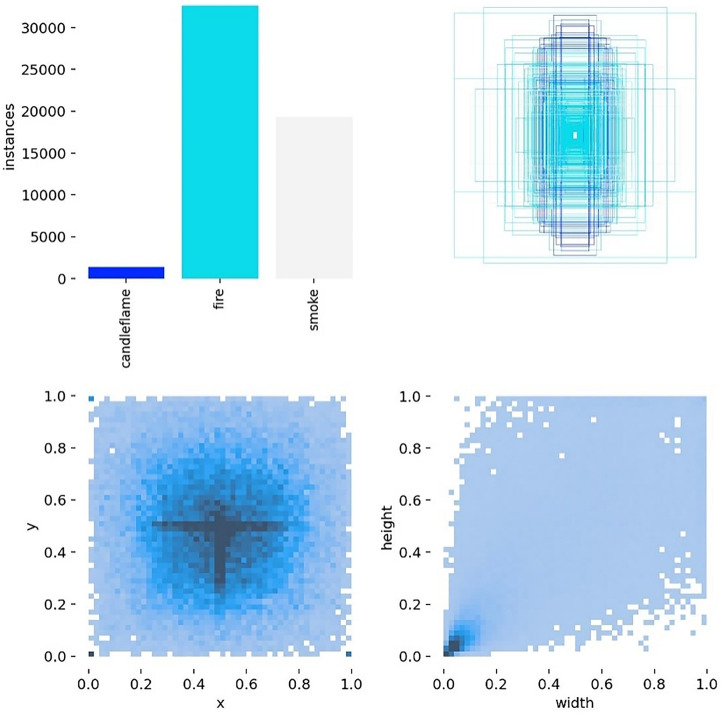
Labels diagram.

According to Fig 12, the image consists of four parts. The first diagram shows the actual data amount for each category label, the second diagram displays the labelled boundary box [24], the third diagram indicates the actual coordinate value of the labelled central point, and the fourth diagram presents the width and height of the labelled matrix. We find that the sum of the data based on the three target types from the photograph is close to 50000.

**Fig 13:**
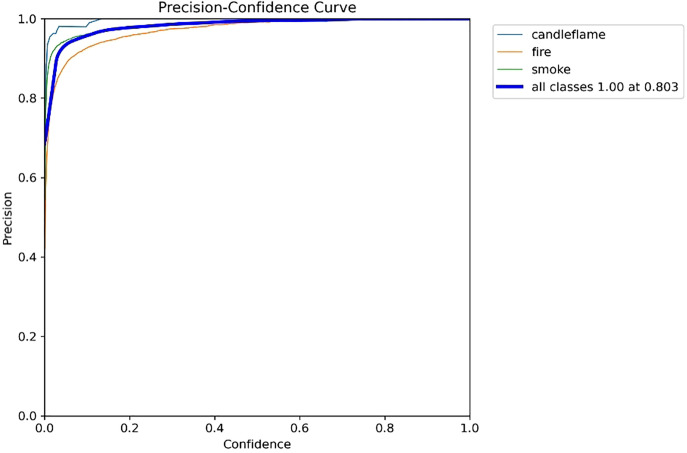
Precision_curve diagram.

As shown in Fig 13, when the number of training rounds reaches 300, the confidence of all types of recognition is approximately 0.803. Additionally, higher confidence correlates with higher accuracy.

**Fig 14:**
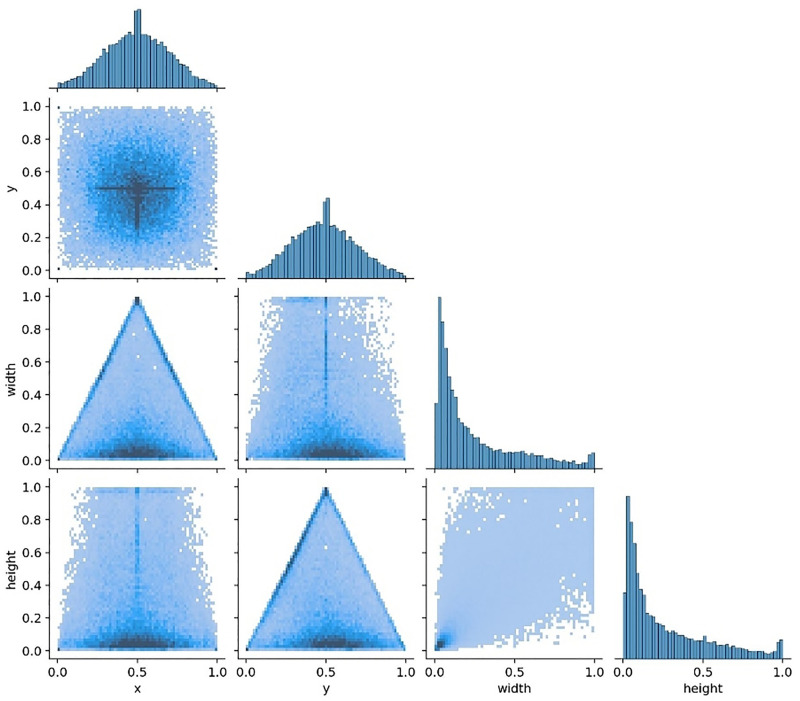
Labels.correlogram.

Fig 14 is a two-dimensional colour matrix graph that illustrates the correlation between the centre point coordinates (x,y) in the predicted label and the width and height of the box. The last graph in each row represents the overall distribution.

### 3.3 Ablation experiment

In order to evaluate the influence of the proposed model strategy on the detection performance, the module ablation method was adopted for experiments, and the optimal design choice was determined. The second group in Table 1 introduces the small object detection layer. The time complexity of this algorithm slightly increases, which leads to a slight decrease in the P of the model. However, this modification improved the performance of small object detection, increasing the Mean Average Precision (mAP) by 0.9%. Therefore, although the increase in the computational load of the model may lead to an increase in time complexity, to a certain extent, this impact can be ignored. In the task of detecting forest fires with small targets, the GAM attention mechanism is of crucial importance. Although its introduction has led to a slight decrease in P to a certain extent, it has improved the detection accuracy of the model. This is because GAM, by learning global importance, can fuse global information, enhance the feature expression of small target areas, make them more prominent in the feature map, and thereby help improve the detection performance of small targets. Overall, with the addition of the small object detection layer and GAM, the P of the model slightly decreased by 13.9%. However, it has increased the mean Average precision (mAP) by 1.4%.

**Table 1:** Results of the model ablation experiment.

Experimental group (YOLOv5l)	Small target detection layer	GAM	Evaluation index			
			mAP50 (%)	P (%)	R (%)	F1-Score(%)
1			91.7	94.2	93.0	89.0
2	√		92.6	91,5	92.0	89.0
**3**	**√**	**√**	**93.1**	**80.3**	**92.0**	**89.0**

Table 1 presents the ablation experiment data of the algorithm in this paper.

### 3.4 Performance comparison of different models

By comparing and analyzing the model with several object detection algorithms as shown in Table 2, the performance of the model in the task of small target forest fire detection was evaluated. mAP50 is a comprehensive indicator for measuring the detection accuracy of a model under different confidence thresholds, reflecting the overall detection ability of the model for multiple types of targets. YOLOv5-PSG led with 93.1% mAP50, an increase of 3.7 percentage points compared to the highest value of the YOLOv3 series (89.4% of YOLOv3-SPP), indicating a significant advantage in its detection accuracy for complex targets. The 5S/m/l of YOLOv were both 91.7%. Although it was slightly lower than that of PSG, it was significantly higher than that of YOLOv3 (88.1%) and YOLOv3-Tiny (80.1%), reflecting the optimization effect of the YOLOv5 infrastructure. The mAP50 of YOLOv3-tiny is only 80.1%, mainly due to the insufficient feature extraction ability caused by the lightweight of the model, and the poor detection effect on small targets or fuzzy targets. YOLOv3-spp enhances the multi-scale feature fusion ability through Spatial pyramid Pooling (SPP), with a mAP50 of 89.4%, but still lags behind the basic version of YOLOv5.

**Table 2:** Results of model comparison tests.

Algorithm	Backbone network	Evaluation index			
		mAP50 (%)	P (%)	R (%)	F1-Score(%)
YOLOv3	DarkNet	88.1	96.8	89.0	86.0
YOLOv3-spp	DarkNet	89.4	99.6	90.0	86.0
YOLOv3-tiny	DarkNet	80.1	91.0	89.0	76.0
YOLOv5s	CSPDarkNet	91.7	94.2	93.0	89.0
YOLOv5m	CSPDarkNet	91.7	94.2	93.0	89.0
YOLOv5l	CSPDarkNet	91.7	94.2	93.0	89.0
**Ours**	**CSPDarkNet**	**93.1**	**80.3**	**92.0**	**89.0**

Table 1 presents the comparative data of the algorithms used in this paper.

The trade-off between accuracy (P) and recall rate (R) shows that the YOLOv3-spp (99.6%) model has an extremely low false detection rate under strict confidence screening, but the recall rate is only 90.0%, posing a risk of missed detection. The recall rate of YOLOv5s/m/l reaches 93.0%, and that of YOLOv5-PSG is 92.0%, indicating that its “missed detection rate” for the target is low. Even if there are a few false detections, they can be filtered through subsequent algorithms. The accuracy (96.8%) and recall rate (89.0%) of the traditional YOLOv3 are relatively balanced, but its overall performance is comprehensively surpassed by YOLOv5. F1-Score is the harmonic mean of accuracy and recall rate, reflecting the overall balancing ability of the model. The YOLOv5 series (n/s/m/PSG) are all 89.0%, indicating that it has achieved the optimal balance between accuracy and recall rate, and is particularly suitable for general detection tasks with high requirements for comprehensive performance. Although both YOLOv3-SPP and YOLOv3 have an F1-Score of 86.0%, the former relies on extremely high accuracy to lower the recall rate, while the latter limits its overall performance due to insufficient recall rate. YOLOv3-tiny is only 76.0%, which is a significant cost for the lightweighting of the model.

In the YOLO-PSG model of this paper, although the newly added small target detection layer pays more attention to small-scale features and can detect more small targets that were originally missed, improving the recall rate and promoting the increase of mAP50 which is sensitive to the recall rate, it may have feature utilization conflicts with the original detection layer and interfere with the detection features of large and medium targets. It reduces the detection accuracy of large and medium targets and lowers the overall accuracy. Although GAM_Attention can adaptively focus on the image area, assist in the extraction of small target features, improve the recall rate and promote the increase of mAP50, it may overly focus on the features of small targets and ignore the important detail features of large and medium targets, resulting in recognition errors and reduced accuracy. The main task of object detection in this paper is to conduct preliminary predictions of forest and mountain fires through high-altitude unmanned aerial vehicles (UAVs), and it is more hoped to effectively detect the early images of mountain fires. Although the reduced accuracy of the model will determine many non-fire data as fires, compared with the possible losses caused by mountain fires, the losses brought by these redundant judgments are much smaller.

## 4 Conclusions

This project involved optimisation research on high-altitude physiological fire source policy based on image recognition. The test results indicate that it can optimise the image recognition of high-altitude forest fire source detection by improving the image quality of datasets and gradually increasing the number of training rounds, ultimately selecting the optimal number of training rounds as 300. This selection is necessary due to the limited data and resources available for high-altitude forest fires on the internet, necessitating the filtering of dataset selection; therefore, only relevant parts of images of fire sources are included in datasets. In terms of the protection of high-altitude forest fire sources, real-life scenarios of these sources present challenges in identifying security risks. In the future, large, high-quality smoke datasets from high-altitude fire sources should be selected to ensure the use of a variety of complex scenes involving small fire sources at high altitudes and diverse smog datasets under different weather conditions. This approach will ensure varying image sizes within the datasets and facilitate multiscale training, thereby enhancing the testing capability of the modules and increasing the confidence level of recognition by optimising the number of rounds of training. Additionally, the robustness [25] and generalisation ability of the model [26] can be improved by rotating, translating and scaling the training datasets [27] while increasing the number of samples. High-altitude research based on image recognition is very important for advancing the field of fire protection in the future. The potential impact of breakthroughs in this area should not be underestimated.
